# Global trends and hotspots of adolescent eating disorders: a bibliometric study and visual analysis

**DOI:** 10.3389/fpsyt.2025.1608930

**Published:** 2025-07-23

**Authors:** Qian Ye, Yiling Yang, Yue Qi, Jiale Li, Huijuan Lei, Lina Sun, Jie Zhang

**Affiliations:** ^1^ School of Nursing, Guangdong Pharmaceutical University, Guangzhou, Guangdong, China; ^2^ Department of Child & Adolescence Psychology, The Zhongshan Third People’s Hospital, Zhongshan, Guangdong, China

**Keywords:** adolescent, eating disorders, bibliometrics, VOSviewer, Citespace

## Abstract

**Background:**

Adolescent eating disorders represent a category of mental illness, marked by elevated mortality and morbidity rates and a significant adverse impact on the quality of life of both patients and their families. As one of the most important directions in eating disorders, adolescent eating disorders have significant research value, social value, and practical significance. Surprisingly, the overall research landscape in this domain has yet to be systematically explored through bibliometric analysis. Consequently, this study aims to employ bibliometric methods to analyze published literature, providing a comprehensive and systematic summary of the current research advancements and hotspots in the field of adolescent eating disorders, as well as to forecast potential research directions and future trends.

**Methods:**

We retrieved studies related to adolescent eating disorders from the Web of Science Core Collection database, covering the period from January 1, 2011, to December 31, 2024. We conducted a bibliometric analysis of the literature, employed the visualization tools CiteSpace and VoSviewer. Our study focused on the number of publications, countries, institutions, journals that have cited the works, references, authors, and keywords, to uncover patterns and trends in the field.

**Results:**

A total of 1,077 articles were retrieved. The number of publications in this field has shown a steady upward trend. The United States leads in adolescent eating disorder research. The University of California is the most productive institution, with 134 papers. The International Journal of Eating Disorders is the most cited journal, with 993 citations. The reference “*American Psychiatric Association, Diagnostic and Statistical Manual, 5^th^*” has the highest citation frequency. Le Grange Daniel is the most prolific author. High-frequency keywords include “prevalence”, “weight”, and “risk factors”. Emerging keywords are “mental health”, “emotion management”, “social media”, and “validation”.

**Conclusions:**

Adolescent eating disorders are gaining increasing global attention. Presently, research on this issue focuses on comorbidities between adolescent eating disorders and other mental illnesses, and their etiology, risk factors, and clinical assessment. In the future, the core research directions in this field will center on verifying the long-term effectiveness of eating disorder treatments, refining personalized assessment and treatment models, and furthering interdisciplinary integration in interventional approaches.

## 1 Introduction

Eating disorders are a range of psychological conditions characterized by abnormal eating behaviors and an excessive preoccupation with weight and body shape (1). These disorders can lead to severe health problems (2). According to the Diagnostic and Statistical Manual of Mental Disorders, Fifth Edition (DSM-5) (3), the main types of eating disorders include anorexia nervosa (AN), bulimia nervosa (BN), binge eating disorder (BED), avoidant/restrictive food intake disorder (ARFID), pica, and rumination disorder. These conditions often emerge during adolescence or early adulthood (1). Globally, the lifetime prevalence of eating disorders in adolescents and young adults is between 0.6% and 26.7% (4). A 2011 US-based cross-sectional survey of adolescents found that the prevalence of anorexia nervosa, bulimia nervosa, and binge-eating disorder was 0.3%, 0.9%, and 1.6%, respectively (5), and these conditions were more common in females than in males (6). Many risk factors for eating disorders in adolescents have been identified, including age, gender, BMI, weight, mental health, and family and social environment. This is likely because adolescents are in a critical stage of physical and psychological development. Their cognitive control is not fully mature, and their emotional regulation mechanisms are relatively fragile, making them more vulnerable to eating disorders than other age groups (7). In addition, eating disorders can lead to serious consequences, such as non - suicidal self - injury, suicidal ideation, and suicidal behavior (8, 9). Recent studies have found a significant link between the duration of eating disorders and an increased risk of early-onset executive dysfunction, particularly in the core areas of decision-making, inhibitory control, and cognitive flexibility. This impairment can undermine the daily functioning of individuals with eating disorders, thereby increasing the risk of self-harm and suicide (10). Furthermore, SJ and colleagues’ research shows that among adolescents with bulimia nervosa and binge-eating disorder, 34% to 53% have experienced suicide attempts during their illness (11). Eating disorders can also lead to other health problems, such as depression, obesity (12), anxiety, OCD, diabetes (13), and renal failure (14). For adolescents with eating disorders, these problems mainly affect their mental and physical well-being. Cumulative research has found that adolescents with eating disorders are more likely to develop mental health problems such as depression, anxiety, and OCD later in life (15–17). It should also be noted that eating disorders can co-occur with other mental disorders, like depression, and worsen depressive symptoms. Currently, psychotherapy is the main treatment for adolescent eating disorders. However, due to economic and geographical factors, only a small number of patients receive effective treatment. In summary, eating disorders in adolescents can significantly impact their academic performance, daily life, and overall development. Therefore, exploring adolescent eating disorders is a significant research direction.

In recent years, adolescent eating disorders have gradually attracted extensive attention from the academic community, with research spanning an increasingly broad range of fields. This issue has expanded beyond the traditional realm of psychiatry to encompass multiple disciplinary dimensions, including psychology and sociology. A significant portion of studies, including several systematic reviews, have conducted in-depth explorations into the prevalence of adolescent eating disorders (18), as well as their risk factors, diagnosis (19), assessment (20), and treatment (21). However, existing studies often focus on narrow aspects and have failed to provide a comprehensive and systematic examination of the evolving trajectory of research on adolescent eating disorders. Meanwhile, with the rapid increase in relevant literature, researchers are confronted with the growing difficulty of obtaining key information and the challenge of accurately grasping the core conclusions of current research and future directions (22). Therefore, there is an urgent need for innovative methods to integrate the knowledge system of published studies, thereby fully and objectively analyzing the knowledge evolution and thematic trends in the field of adolescent eating disorders. Bibliometric analysis is a statistical method for analyzing literature that includes both qualitative and quantitative assessments. It can cluster and analyze detailed information of a specific research field, such as the number of publications per year, countries, institutions, journals, authors, and keywords. This method is capable of revealing the knowledge structure of a particular academic domain. By employing techniques such as data mining, information processing, statistical analysis, and mapping, it visualizes the evolution of knowledge communities. This helps scholars understand the academic achievements in their research area and identify future research directions (23, 24). Numerous bibliometric analyses focusing on adolescent populations have been conducted globally (25), highlighting the significant role of bibliometrics in this field. For instance, studies have examined issues such as adolescent insomnia (22), non - suicidal self – injury (26), and the comorbidity of overweight/obesity and depression in children and adolescents (27). However, to date, no bibliometric analysis has specifically investigated publications related to adolescent eating disorders. Therefore, we aim to apply the same methodology to conduct a bibliometric review of this domain. Through this comprehensive and objective synthesis and summary of the current research landscape, hotspots, and developmental trajectories, we hope to provide a valuable reference for researchers interested in this field.

Our primary aim is to conduct a comprehensive scientometric analysis of the evolving research trends in adolescent eating disorders over the past decades by using co-occurrence keyword networks. Our secondary objective is to provide researchers with a set of metrics and performance analyses for collaborative networks (including countries, institutions, authors, journals, references, and keywords).

## 2 Methods

### 2.1 Data sources

The literature data analyzed in this study were sourced from the Web of Science Core Collection (WoSCC) database, which is widely recognized internationally (28). This database was selected for its authoritative and comprehensive nature in the fields of bibliometrics and literature visualization (29). The systematic data collection approach employed by WoSCC has been widely acknowledged and applied in numerous studies (30). Additionally, to avoid bias caused by daily updates to the database, all literature searches and downloads for this study were completed on the same day (all data were retrieved on March 12, 2025).

### 2.2 Search strategy

Literature was retrieved online from the WoSCC database, and the retrieval process yielded 3,040 results for the period from January 1, 2011, to December 31, 2024. The search query used was TS = ((“adolescen*” OR “teen*” OR “youth*” OR “young*” OR “juvenile*” NOT “adult”) AND (“eating disorder*” OR “bulimia nervosa” OR “binge eating” OR “anorexia nervosa”)).

### 2.3 Inclusion/exclusion criteria

**Figure 1:**
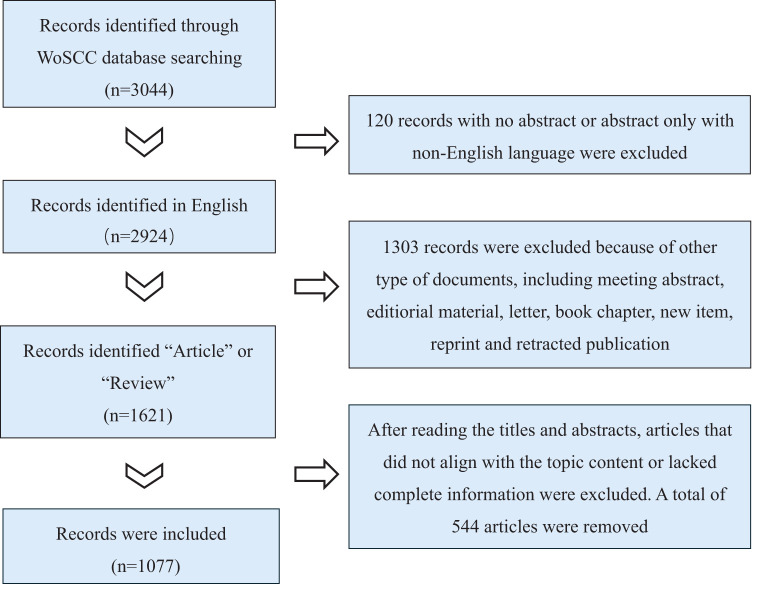
Flow chart of literature screening.

Two researchers independently screened the literature by reading titles and abstracts, cross-validated the results, and excluded studies unrelated to adolescent eating disorders. Disagreements were resolved through discussion with the first author. Inclusion criteria: 1) focus on adolescents with eating disorders; 2) English language; 3) article or review type. Exclusion criteria: 1) mismatched topic or population; 2) conference papers, abstracts, or duplicates. All records meeting the inclusion and exclusion criteria were exported, converted into plain text files named in the “download_XXX.txt” format, and imported into CiteSpace (version 6.2.R3) and VOSviewer (version 1.6.20) for analysis. After manual screening and deduplication in CiteSpace, 1,077 English-language papers were obtained (**Figure 1**).

### 2.4 Analysis tool

CiteSpace is a visualization tool developed by Professor Chaomei Chen (31). In this study, CiteSpace was used to conduct co-citation analyses of countries, institutions, and authors, as well as co-occurrence analyses of cited journals, references, and keywords. Additionally, cluster and burst analyses of keywords were performed. The specific parameter settings are as follows: period (from January 2011 to December 2024, with each slice being one year), term sources (titles, abstracts, author keywords, and keyword extensions), pruning (Pathfinder), node types (authors, institutions, countries, keywords, references, and cited journals), and other parameters are set as default (link retention factor: LRF = 3.0; e - value: e = 1; look - back years: LBY = 5; strength: cosine; range: within - slice; g - index: k = 25; top N items: 50; top N%: 10.0%; maximum number of items per slice: 100; visualization: cluster view - static and display merged network). CiteSpace integrates key information such as burst detection, betweenness centrality, and co-occurrence networks, which can be used to visualize the current state, hotspots, and frontier areas of research. In different co-occurrence maps, nodes represent countries, institutions, authors, cited journals, references, and keywords. The size of the nodes reflects their frequency of occurrence or citation, the color of the nodes indicates the year of occurrence or citation, and the lines between nodes signify collaborative or co-citation networks (32). The burst detection algorithm is an effective tool for capturing a sharp increase in citations and keywords over a specific period. In this mapping, the blue line represents the time interval, while the red line indicates the period during which the burst of citations and keywords occurs.

VOSviewer, developed by the University of Leiden in the Netherlands (33), was used in this study to construct network maps of authors and keywords. Nodes in these maps represent authors and keywords, with their colors distinguishing different clusters within the network. Various clusters symbolize potential research groups in author collaboration networks or keyword clusters. The width of lines between nodes indicates the degree of author collaboration or keyword co-citation. The data retrieved were imported into VOSviewer in plain text format. Parameter settings: minimum threshold of 15 publications for authors and 13 occurrences for keywords.

Microsoft Office Excel 2019 was used to create annual publication trend charts for different countries and to generate tables for the information needed in this article, including data on productive countries, institutions, cited journals, references, authors, and keywords.

## 3 Results

### 3.1 Annual global publication outputs

**Figure 2:**
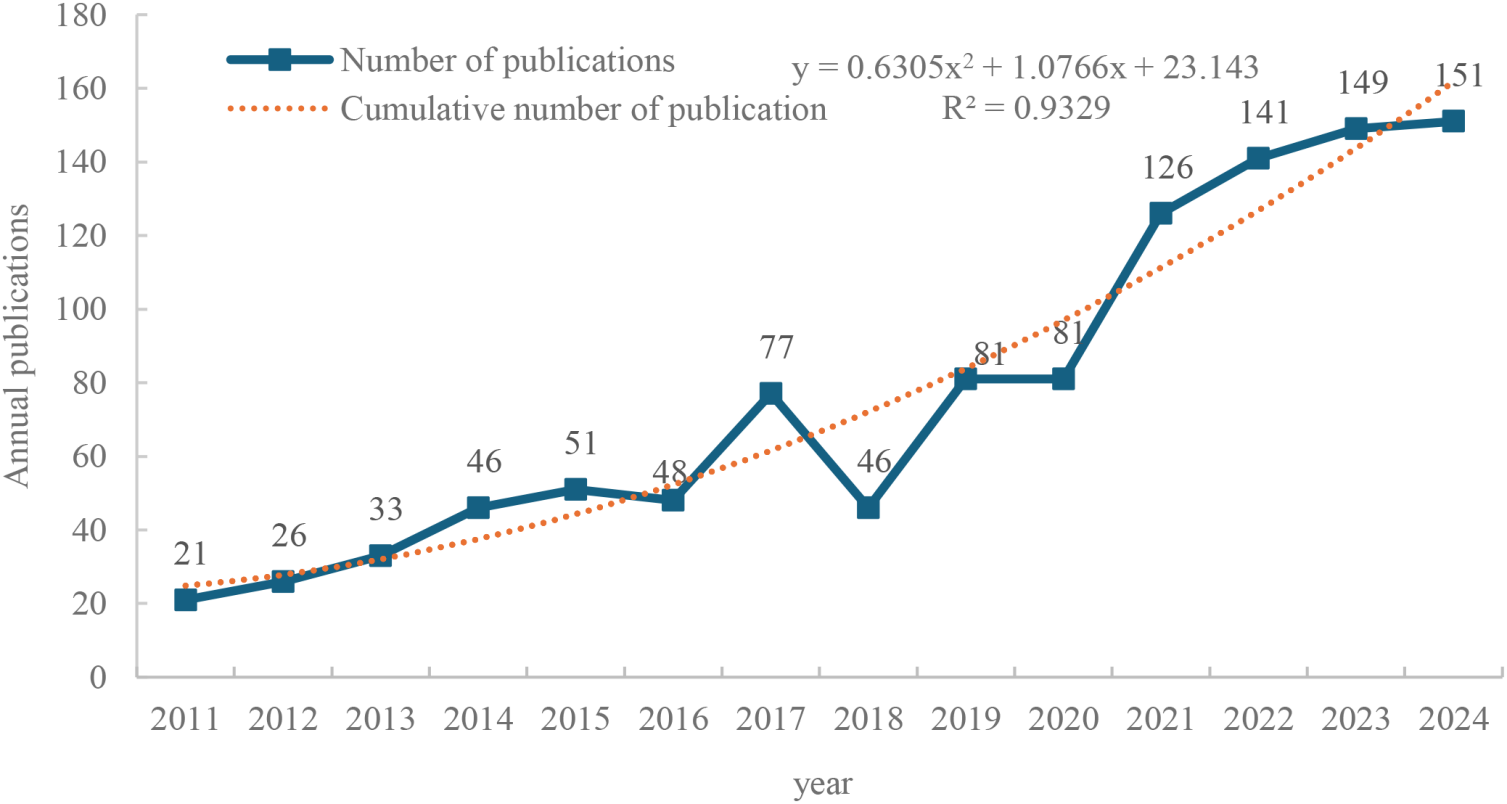
Number of publications on adolescent eating disorders by year.

A total of 1,077 English-language articles were included in this study, as shown in **Figure 2**. The number of publications increased from 21 in 2011 to 151 in 2024. Although the growth trend of publications from 2011 to 2020 exhibited minor fluctuations, the annual publication volume remained below 100 articles during this period. It was not until 2021 that there was a noticeable increase in publication volume. The annual increase in publications from 2021 to 2024 was much more substantial than in the preceding years, suggesting that adolescent eating disorders in the post-Coronavirus Disease 2019 (COVID-19) era have become one of the emerging research hotspots among scholars. In addition, Microsoft Excel was employed to visualize the annual number of published papers, with a trend line added to forecast future publication volumes. The predictive growth model is represented by the equation y = 0.6305x² + 1.0766x + 23.143, where x denotes (forecast year - 2011) and y represents the projected annual number of publications. Moreover, the curve of function fitting indicates a significant correlation between the annual growth trend and the years of publication output (R²= 0.9329), suggesting that the number of future studies in this field will continue to rise.

### 3.2 Country analysis

**Figure 3:**
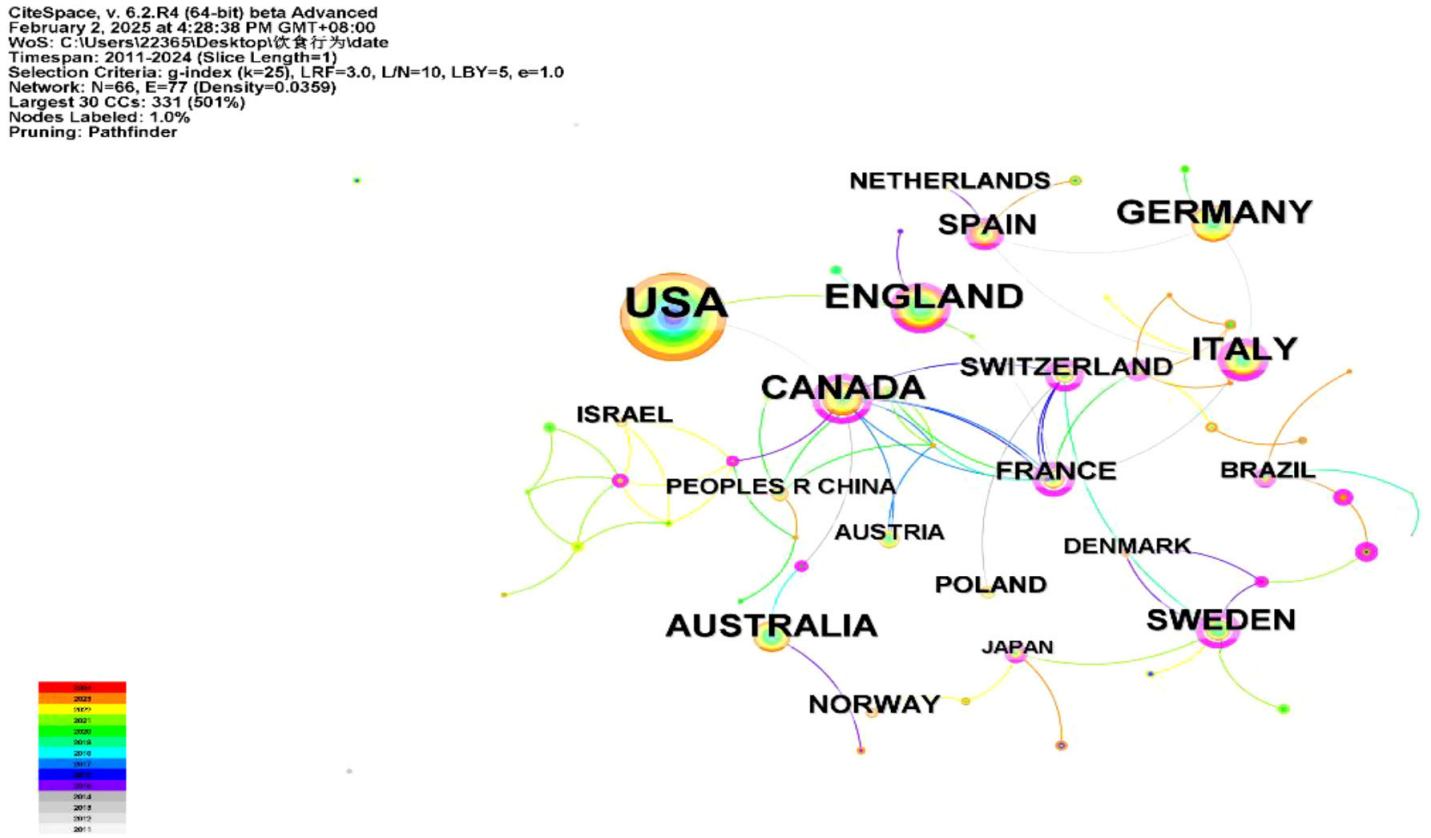
Country network co-occurrence map.

We used CiteSpace to generate a national co-citation network map (**Figure 3**). In the map, circles symbolize countries, with larger circles indicating more publications. The lines between circles represent connections between countries. The map contains 66 nodes and 77 links, with a network density of 0.0359, showing frequent international collaboration. Betweenness centrality, marked by purple rings whose thickness denotes the level of centrality, reflects a country’s research significance. The US led the research with 461 papers (43.36%). England and Germany ranked second and third, with 142 (13.18%) and 117 papers (10.86%). France (0.90), Switzerland (0.79), and Sweden (0.72) had the highest centrality, indicating their authority and prominence in the field (**Table 1**).

**Table 1:** The top 15 productive countries.

Ranking	Country	Publications	Centrality
1	USA	461	0.05
2	England	142	0.30
3	Germany	117	0.05
4	Canada	115	0.65
5	Australia	100	0.05
6	Italy	95	0.36
7	Sweden	51	0.72
8	Spain	48	0.19
9	France	34	0.90
10	Norway	33	0.00
11	Switzerland	31	0.79
12	China	25	0.09
13	Netherlands	24	0.00
14	Denmark	23	0.00
15	Brazil	22	0.05

### 3.3 Institutional analysis

**Figure 4:**
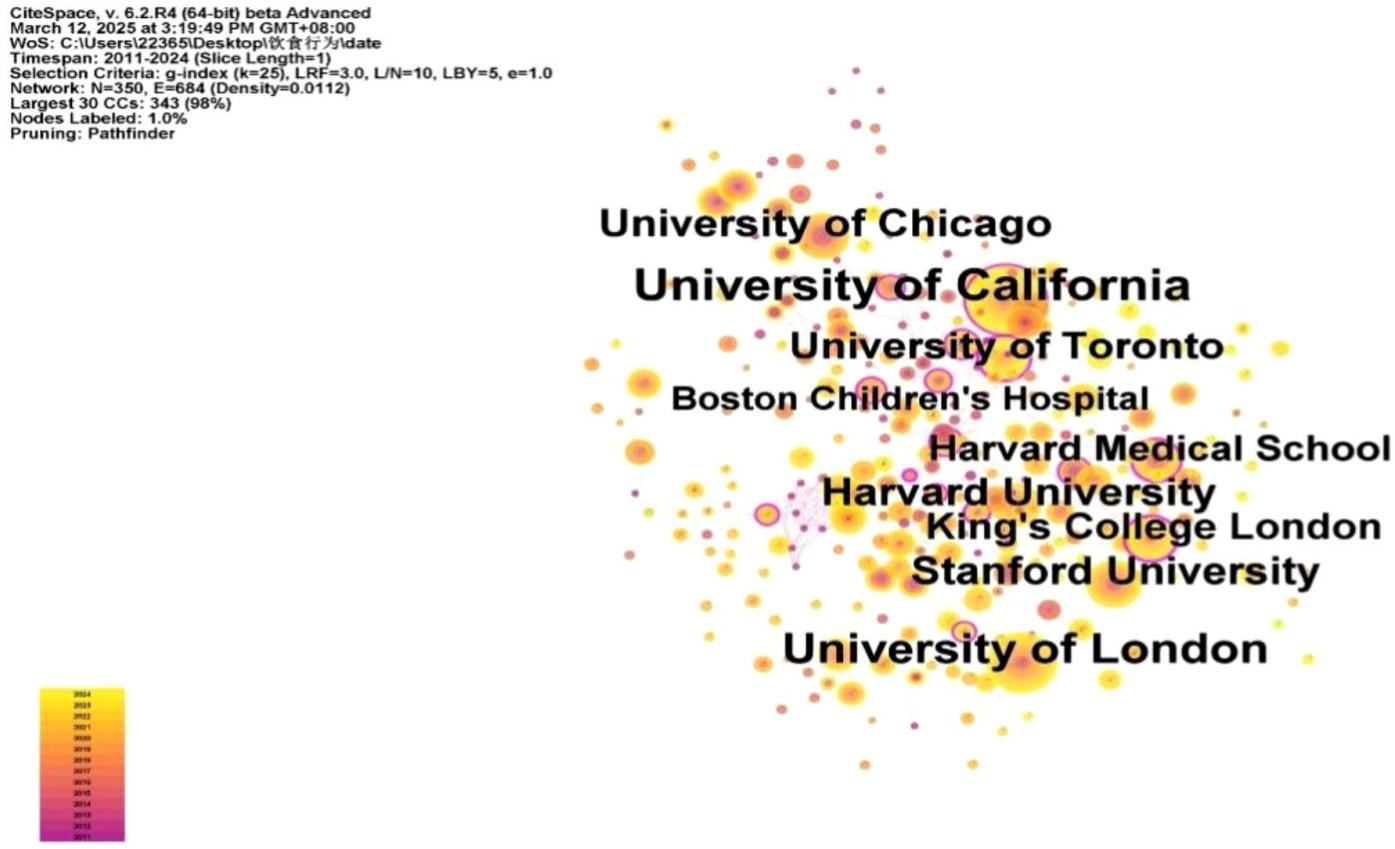
Institutional network co-occurrence map.

**Figure 5:**
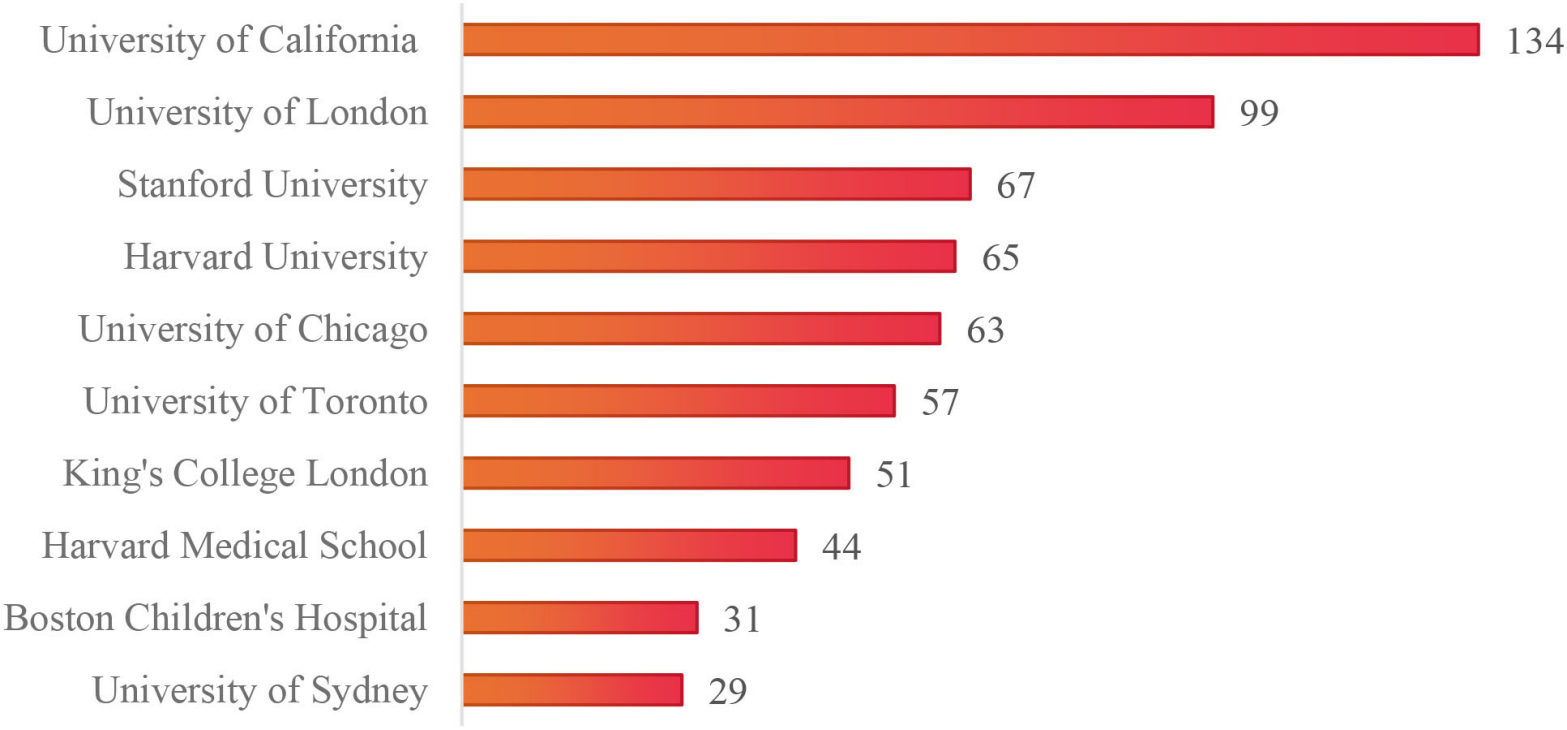
Top 10 Institutions in terms of the number of publications.

This study encompassed 343 research-related institutions (**Figure 4**). The network had 343 nodes and 1,891 links, with a density of 0.0322, indicating close institutional cooperation. Institutions such as universities and specialized pediatric and adolescent hospitals are key players in adolescent eating disorder research. The University of California tops the list with 134 papers (12.44%), followed by University College London (99, 9.19%) and Stanford University (67, 6.22%) (**Figure 5**). The University of California and University College London have formed a stable recent collaboration, focusing on child and adolescent mental illness and neurodevelopment. The former emphasizes neuroscience and technological aspects (e.g., brain-computer interfaces), while the latter integrates epidemiology and psychological theories. Spatially, the institutions are mainly in economically developed European and American countries, with few in developing nations, which limits the research scope but also offers new directions for future studies.

### 3.4 Cited journal analysis

**Figure 6:**
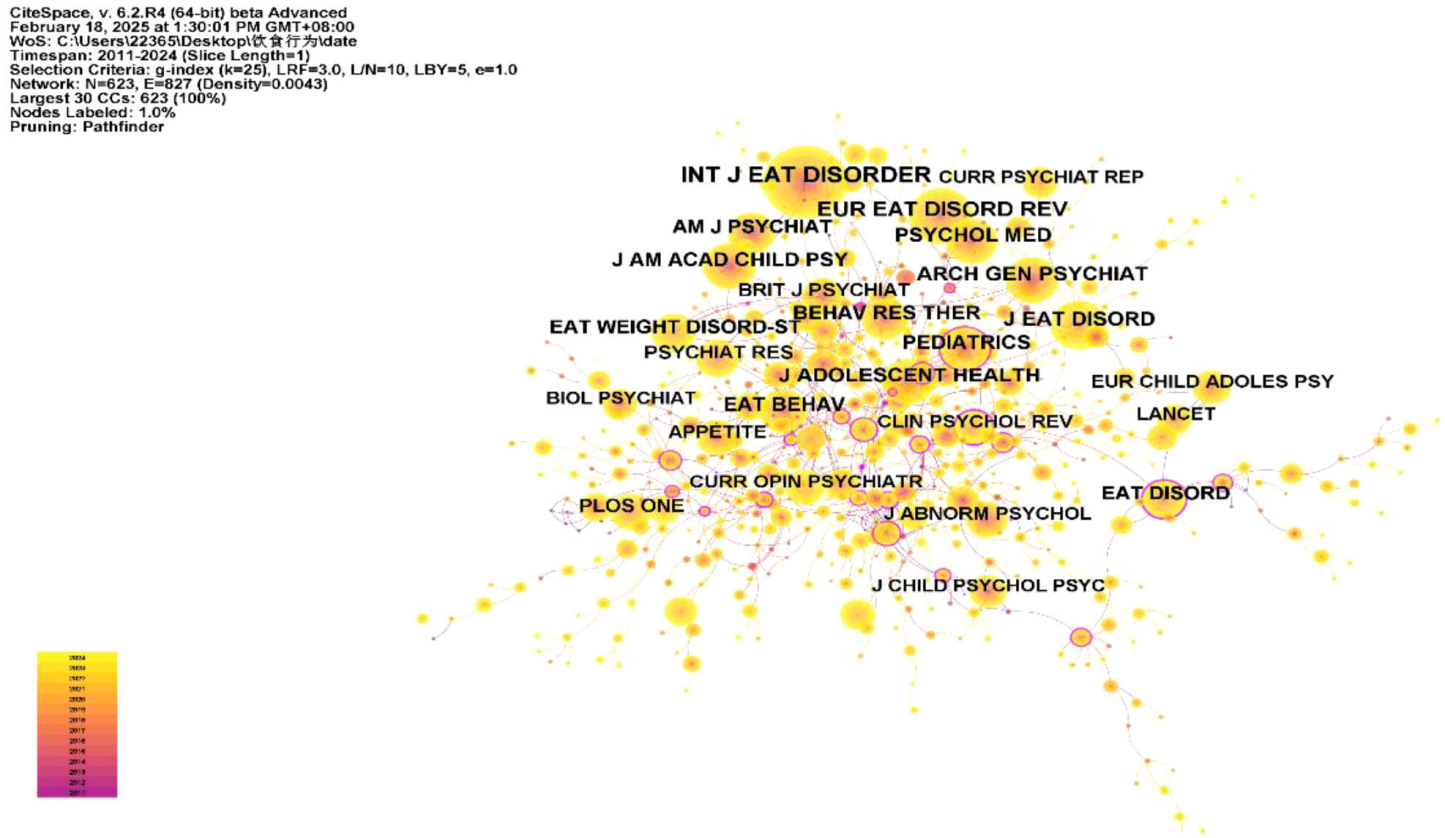
Cited journal network co-occurrence map.

We used CiteSpace to conduct a co-occurrence analysis of journal citations (**Figure 6**). The top 10 cited journals are shown in **Table 2**. The most-cited journal is the *International Journal of Eating Disorders* (n=993), followed by *European Eating Disorders Review* (n=629) and *Journal of Adolescent Health* (n=456). These journals are all classified in the JCR Q2 quartile in the 2023 Journal Citation Reports, indicating that the articles they publish are of high quality and that the studies featured in these journals are reliable in terms of their impact and representativeness.

**Table 2:** The top 10 highly cited journals.

Ranking	Cited Journal	Citations	IF (2023)	Centrality
1	*International Journal of Eating Disorders*	993	4.7	0.02
2	*European Eating Disorders Review*	629	3.9	0.05
3	*Journal of Adolescent Health*	456	5.5	0.03
4	*Archives of General Psychiatry*	454	22.5	0.08
5	*Journal of Eating Disorders*	450	3.5	0.03
6	*Psychological Medicine*	433	5.9	0.01
7	*Journal of the American Academy of Child and Adolescent Psychiatry*	428	9.2	0.02
8	*Eating Behaviors*	402	2.4	0.03
9	*Pediatrics*	387	6.2	0.14
10	*Behavior Research and Therapy*	377	4.2	0.01

### 3.5 References analysis

**Figure 7:**
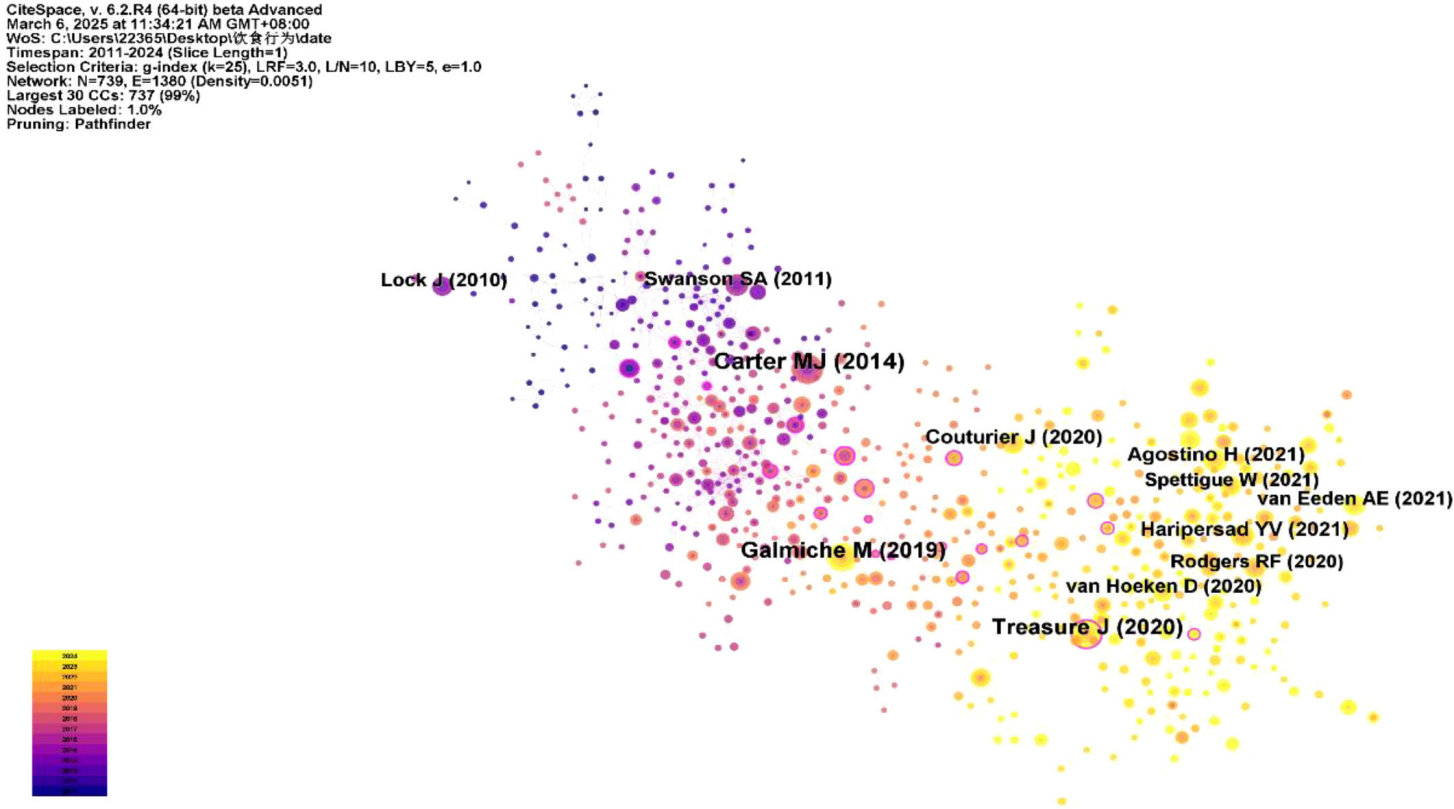
Cited references co-occurrence network map.

The co-occurrence map of references is shown in **Figure 7**, with the top 10 most-cited references listed in **Table 3**. The map includes 739 nodes and 1,380 links. Nodes range in color from deep purple to light yellow, indicating an increasing sequence of publication years, with larger nodes representing higher numbers of publications. Lines signify connections between nodes. The reference “*American Psychiatric Association, Diagnostic and Statistical Manual, 5^th^*” has the highest citation frequency (n=60) (34). The reference with the highest betweenness centrality is the article “*Eating disorders*” published in The Lancet.

**Table 3:** The top 10 co-cited references.

Ranking	Cited reference	Centrality	Frequency
1	American Psychiatric Association, Diagnostic and Statistical Manual, 5th	0.03	60
2	Eating disorders	0.27	48
3	Prevalence of eating disorders over the 2000-2018 period: a systematic literature review	0.02	47
4	Trends in the Incidence of New-Onset Anorexia Nervosa and Atypical Anorexia Nervosa Among Youth During the COVID-19 Pandemic in Canada	0.02	33
5	Outbreak of anorexia nervosa admissions during the COVID-19 pandemic	0.02	32
6	Canadian practice guidelines for the treatment of children and adolescents with eating disorders	0.00	31
7	Prevalence and correlates of eating disorders in adolescents. Results from the National Comorbidity Survey replication	0.00	30
8	Incidence, prevalence and mortality of anorexia nervosa and bulimia nervosa	0.03	28
9	Randomized clinical trial comparing family-based treatment with adolescent-focused individual therapy for adolescents with anorexia nervosa	0.00	27
10	Review of the burden of eating disorders: mortality, disability, costs, quality of life, and family burden	0.05	26

### 3.6 Author analysis

**Figure 8:**
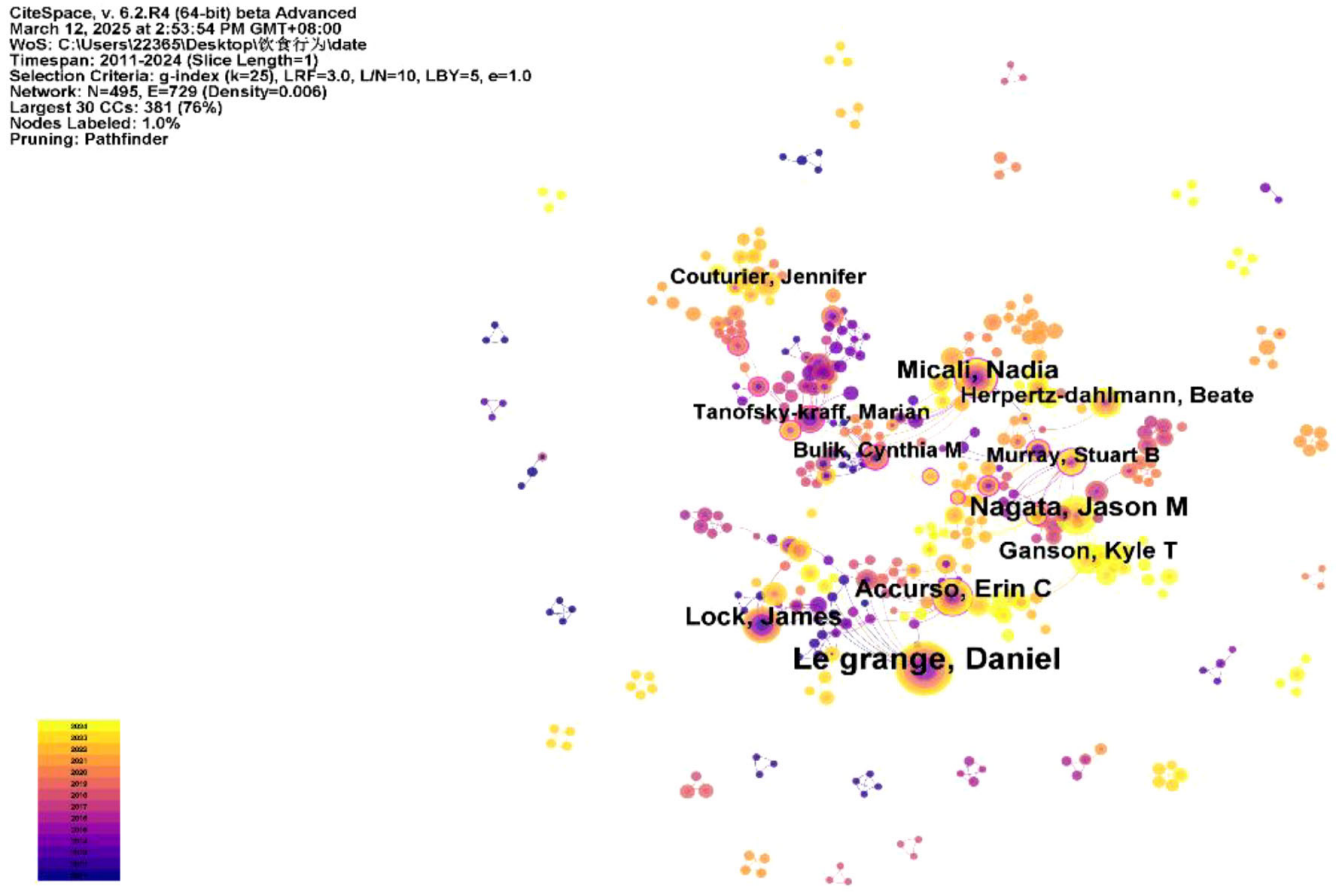
Author co-occurrence network map.

**Figure 9:**
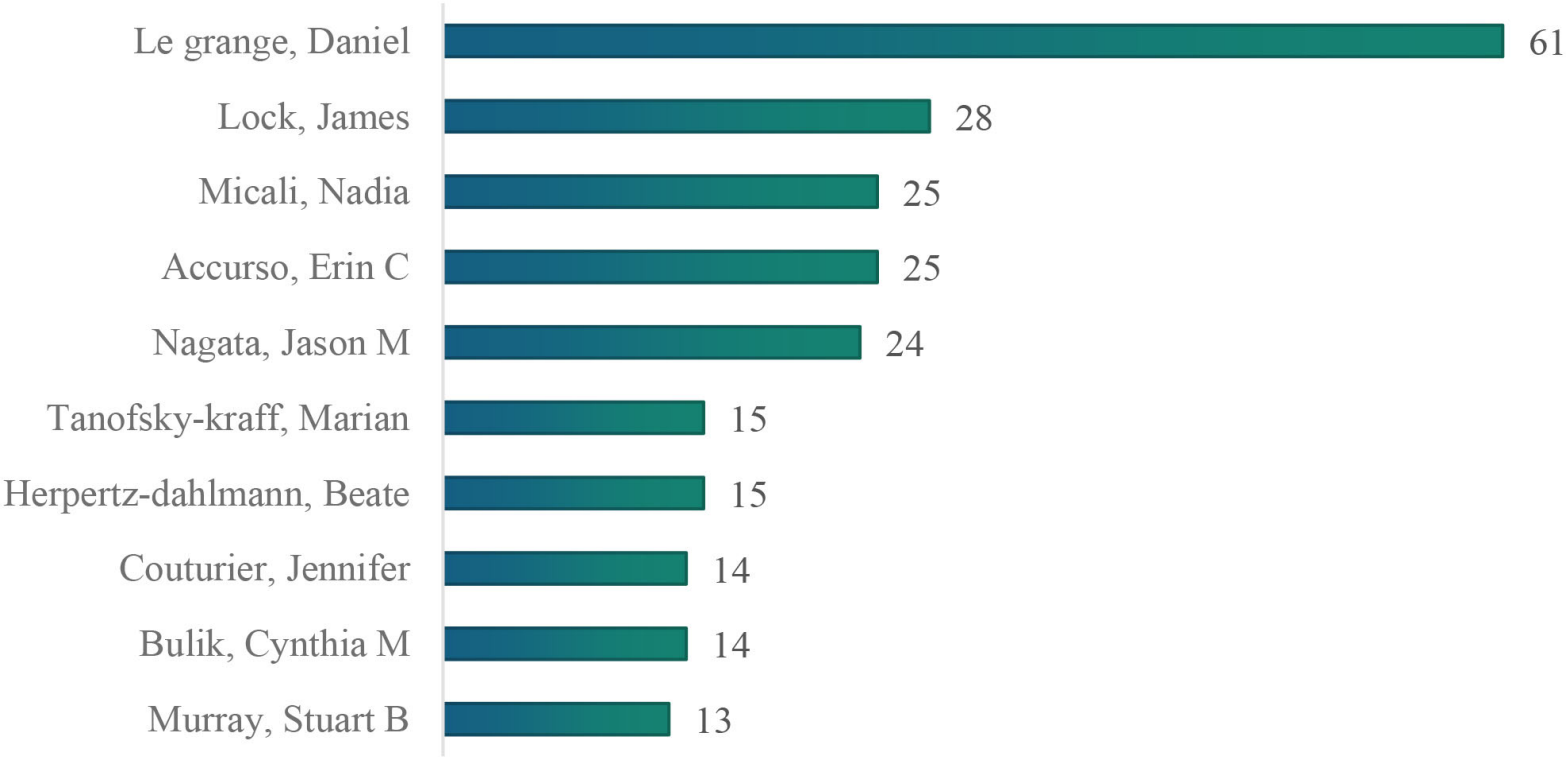
Top 10 authors in terms of the number of publications.

**Figure 10:**
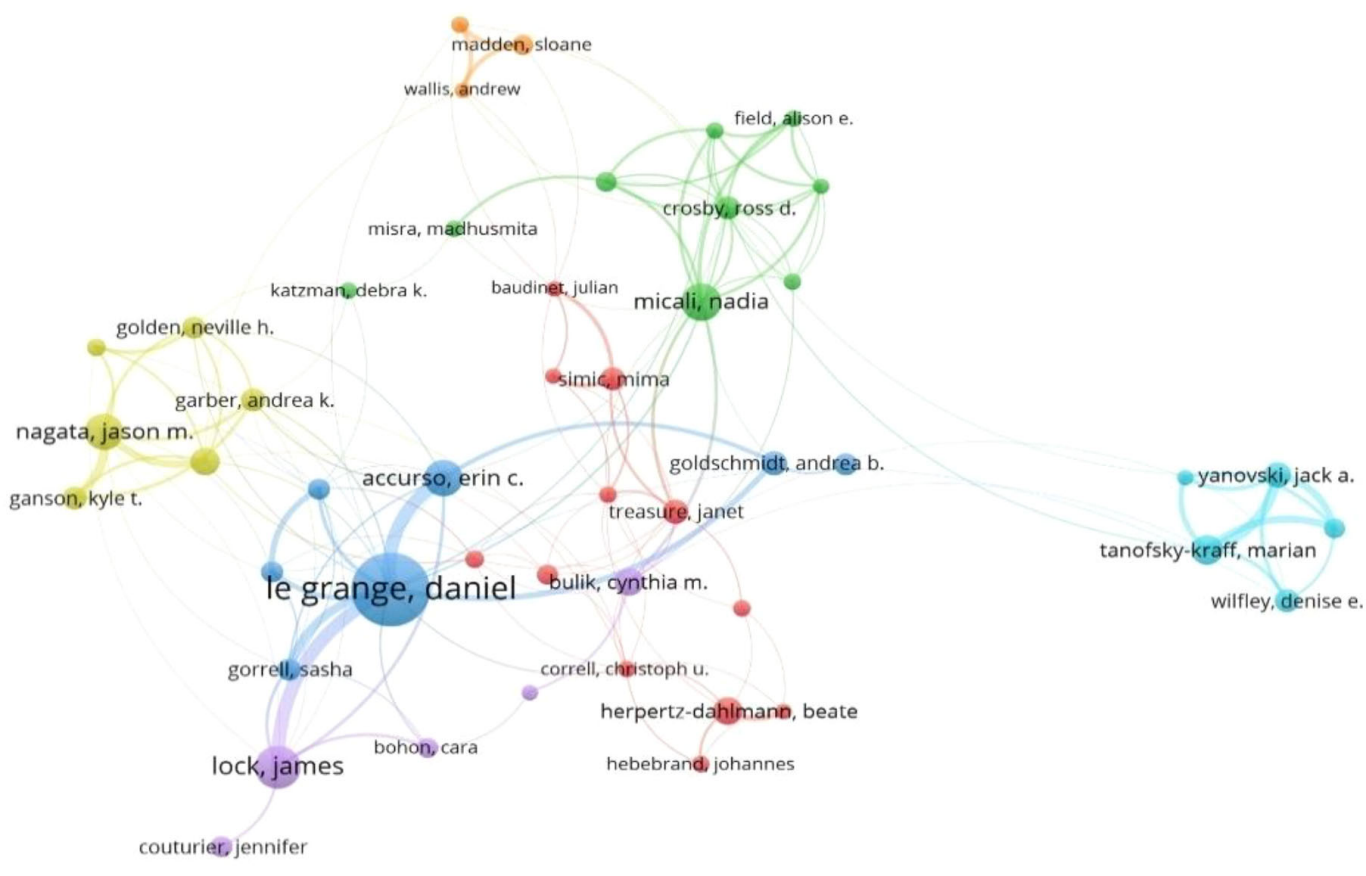
The co-authorship network of authors.

A total of 495 authors contributed to the 1,077 English-language articles. According to bibliometric analysis, the research follows Lotka’s Law, with the majority of authors (72.53%) publishing ≤2 papers, while a small number of authors have contributed the majority of research outcomes. The minimum publication threshold for core authors in this field is calculated as M≈ 0.749×√*Nmax* (where *M* represents the minimum number of publications for influential authors, and *Nma*x is the publication count of the most prolific author, with *Nmax* = 61 in this dataset). Therefore, authors who have published six or more papers are identified as core authors. A total of 57 core authors were identified, who collectively published 564 papers, accounting for 52.36% of the total number of papers, meeting the criterion of Price’s Law that core authors should contribute more than 50% of the total publications. Using CiteSpace for visual analysis, each node represents an author, with larger nodes indicating more published papers. Lines between nodes signify author collaboration, with thicker lines representing closer collaboration. The map shows 495 nodes and 729 links, with a network density of 0.006 (**Figure 8**). Le Grange Daniel is the most prolific author with 61 publications (**Figure 9**), followed by Lock James (n=28), Micali Nadia (n=25), Accurso Erin C (n=25), and Nagata Jason M (n=24). However, the lack of dense connections between authors indicates that research teams in the field of adolescent eating disorders tend to work independently with relatively limited collaboration (**Figure 10**).

### 3.7 Keywords analysis

#### 3.7.1 Co-occurrence analysis

**Figure 11:**
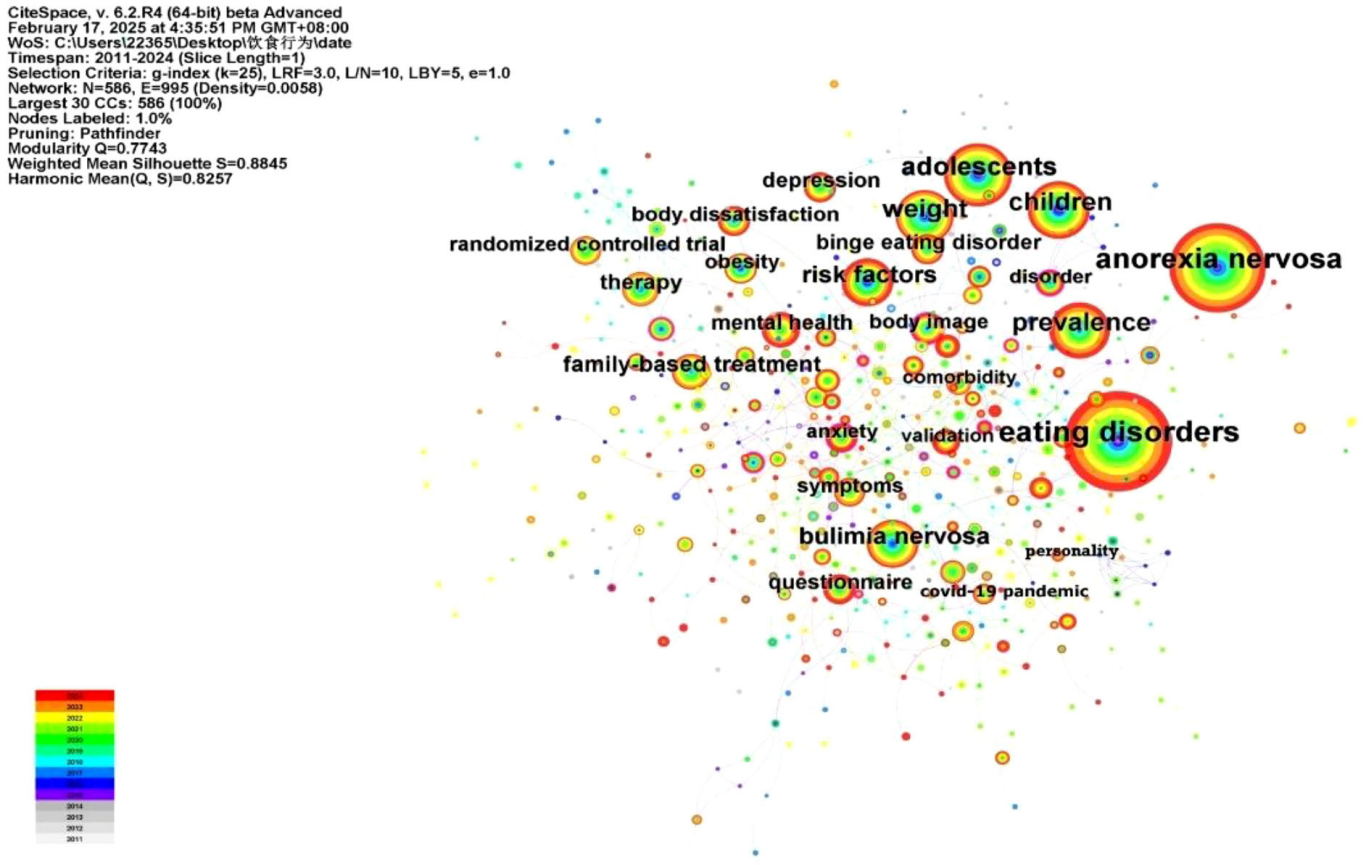
Keyword co-occurrence network map.

**Figure 12:**
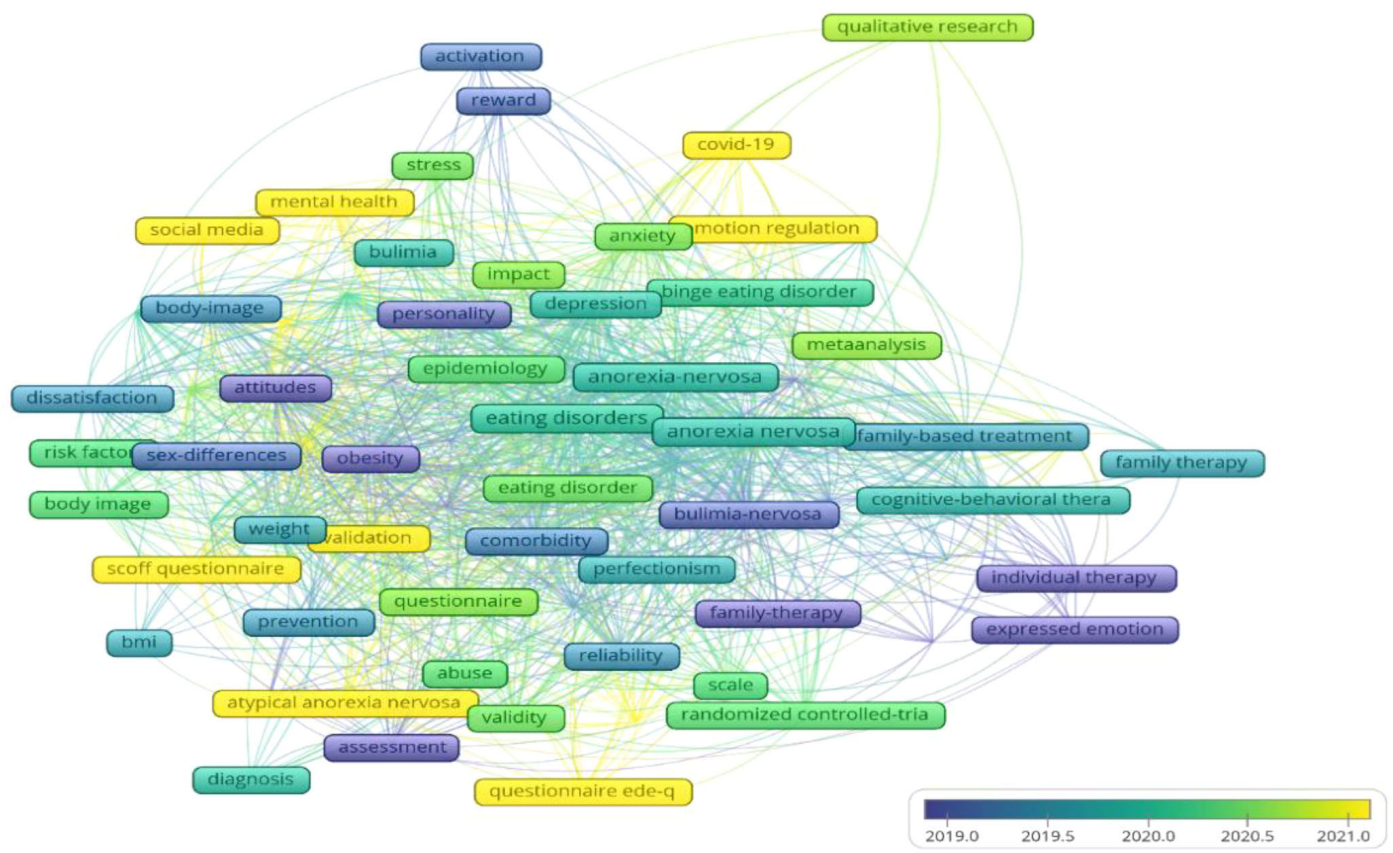
The overlay visualization of high-frequency keywords. From: VOSviewer.

Using CiteSpace, we performed a co-occurrence analysis of keywords related to adolescent eating disorders (**Figure 11**). In the resulting map, there are 586 nodes and 995 links, giving a network density of 0.0058. Each node stands for a keyword, with larger nodes indicating higher keyword frequency. After removing the topic words “eating disorder”, “bulimia nervosa”, “binge eating”, and “anorexia nervosa”, **Table 4** lists the top 10 keywords ranked by frequency and centrality. These keywords cover the etiology, risk factors, comorbidities, assessment, and interventions for adolescent eating disorders. The overlay visualization of keywords is shown in **Figure 12**.

**Table 4:** The top 10 keywords with the highest citation frequency and centrality.

Ranking	Keywords	Frequency	Ranking	Keywords	Centrality
1	Prevalence	255	1	anxiety	0.22
2	Weight	245	2	attitudes test	0.16
3	risk factors	185	3	body mass index	0.15
4	Behavior	177	4	disorder	0.15
5	family-based treatment	135	5	comorbidity	0.14
6	Therapy	115	6	psychopathology	0.13
7	mental health	103	7	body image	0.13
8	Depression	95	8	follow up	0.13
9	randomized controlled trial	92	9	activation	0.11
10	Questionnaire	91	10	gender	0.10

#### 3.7.2 Cluster analysis

**Figure 13:**
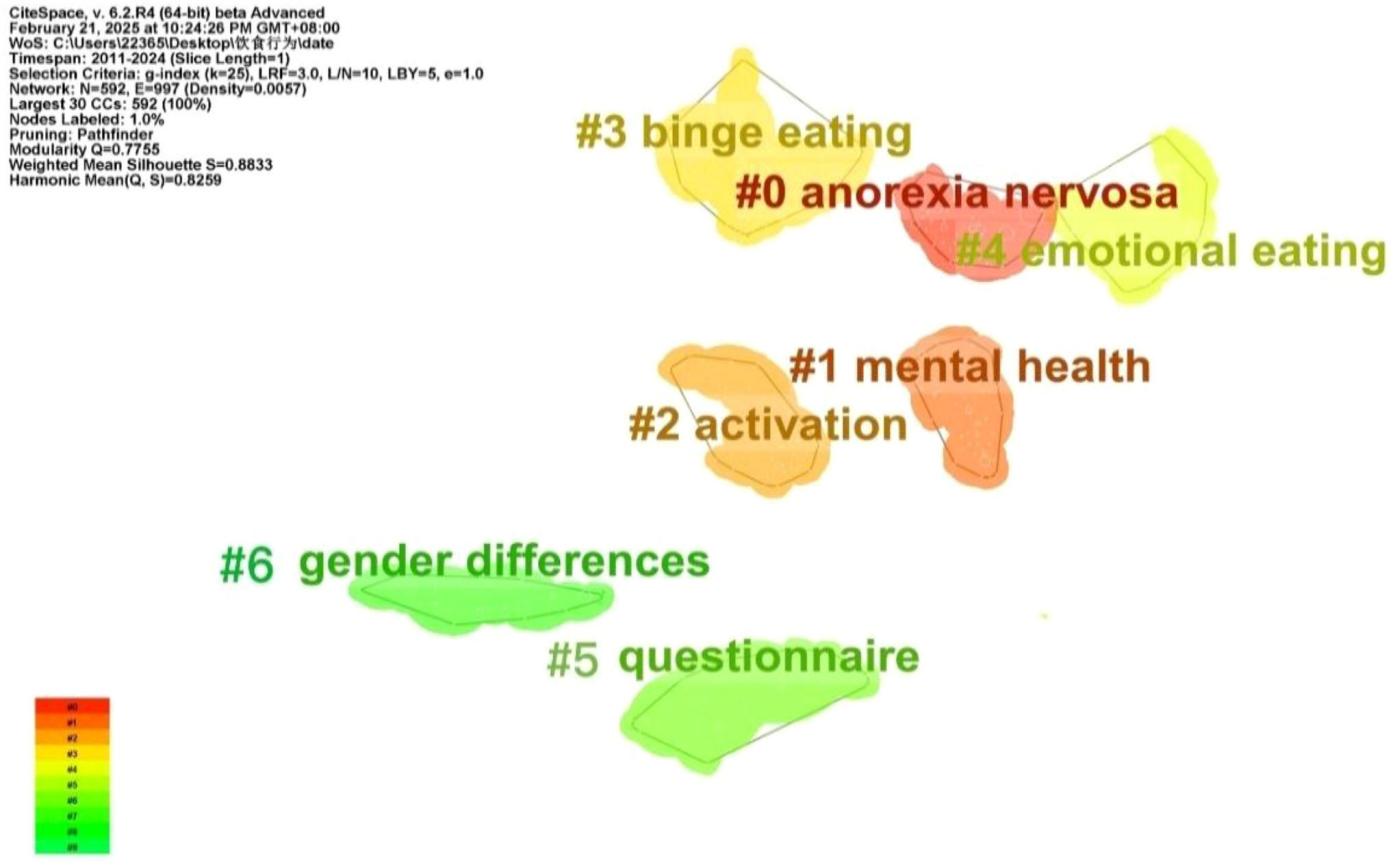
Keyword cluster visualization.

On the basis of the keyword co-occurrence analysis, the likelihood ratio (LLR) algorithm was used to cluster keywords by homogeneity. The cluster analysis map includes 592 nodes and 997 links, with a network density of 0.0057. CiteSpace evaluated the network structure quality and cluster clarity using the modularity value (*Q*) and average silhouette value (*S*). In this study, Q was 0.7755 (*Q* > 0.3 indicates a significant modular structure), and S was 0.8833 (*S* > 0.5 indicates that the clustering results are reasonable). The cluster analysis revealed the distribution of different research areas. Cluster size is inversely proportional to cluster number; larger clusters have smaller numbers. Thus, cluster #0 is the largest (31). In the co-occurrence network of adolescent eating-disorder keywords, seven clusters were identified (**Figure 13**). Cluster #1 “mental health” can assess interventions for such disorders; cluster #5 “questionnaire” shows their assessment methods; clusters #2 “gender difference”, #4 “emotional eating”, and #6 “activation” cover the etiology and risk factors; clusters #0 “anorexia nervosa” and #3 “binge eating” are the main research topics.

#### 3.7.3 Burst detection analysis

**Figure 14:**
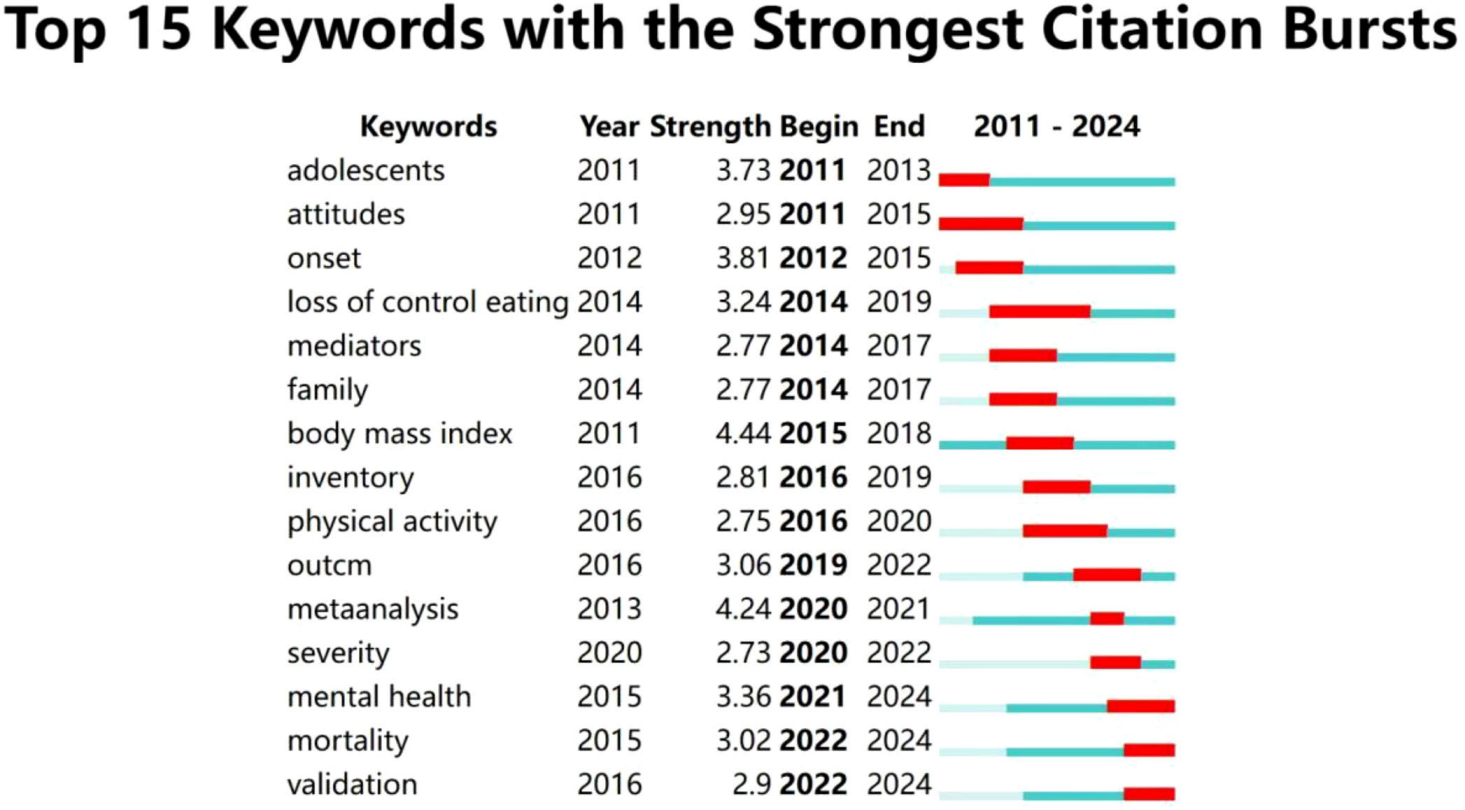
Keyword burst visualization.

Burst detection analysis can quickly identify keywords whose frequency of occurrence has significantly increased during a specific period, helping to reveal hotspots and trends in research fields. As shown in **Figure 14**, “body mass index” has the highest burst strength (4.44), indicating that it was a focal point for researchers from 2015 to 2018. The keyword with the lowest burst strength is “severity”(2.73), with a duration from 2020 to 2022. Looking at the timeline of research, different periods have seen different hotspots in adolescent eating disorder research. The year 2019 was a key turning point. Before 2019, the main focus was on demographic surveys and analyses of adolescent eating disorders. After 2020, the COVID-19 pandemic increased the prevalence and mortality rate of adolescent eating disorders, shifting the research emphasis to the validation of treatments for these disorders. The keywords highlighted in yellow-green and yellow in **Figure 12** mark emerging trends in recent adolescent eating disorder research, such as “social media”, “mental health”, “emotion management”, and “COVID-19”.

## 4 Discussion

### 4.1 Major findings

This study employed visualization tools to analyze 1,077 articles in the field of adolescent eating disorders. The findings revealed that although the number of publications in this field has fluctuated over the past decade, there has been a significant increase since the outbreak of COVID-19. This surge is likely closely related to the containment measures implemented during the pandemic. Specifically, the enforcement of home-quarantine policies led to a substantial reduction in outdoor activities for adolescents. Prolonged periods of solitude and social isolation significantly heightened feelings of loneliness and psychological stress, thereby triggering or exacerbating eating-disordered behaviors (35). These conditions also induced psychological issues such as fear, anxiety, and loneliness among adolescents and increased their risk of depression. Consequently, adolescent eating disorders garnered considerable attention, resulting in a dramatic increase in research output and reaching an all-time high for the first time in 2021.

Visualizations of countries and institutions show that developed countries and institutions, represented by the US, have the highest publication output in eating disorder studies. This can be attributed to the following two factors. Firstly, diagnostic criteria for mental disorders, which are largely influenced by local psychiatric schools, originate primarily from high-income countries like the US, which leads to higher prevalence of reported rates in developed countries. Secondly, Western countries, including the US, have high GDP, abundant funds, advanced equipment, and a strong research system, which facilitates multidisciplinary collaboration and provides important platforms for global academic exchange. Moreover, the participation of developing countries is increasing, although country co-occurrence networks show that the burden of adolescent eating disorders is concentrated in high-income developed countries. Previous literature indicates that the burden of eating disorders is growing across all socio-economic index regions, particularly in developing countries (36). This trend suggests that future research focus may shift from developed to developing countries. This highlights the need for resource planning and prioritization of health policies. It also suggests that future efforts should strengthen cooperation and exchange between developing and developed countries to jointly address the global challenge of eating disorders in adolescence (37).

In terms of journal citations, the *International Journal of Eating Disorders* is the most cited (n=993). The top 10 journals mainly focus on pediatrics, psychology, and psychiatry but have relatively low impact factors, indicating that research quality in this field needs to be improved. The citations of articles in this field are increasing exponentially, with the top 10 articles each cited over 300 times. The American Psychiatric Association’s Diagnostic and Statistical Manual of Mental Disorders, 5th Edition, has the highest citation frequency. This manual provides detailed diagnostic criteria for eating disorders and other mental disorders (34). Second is the 2020 Lancet paper “*Eating disorders*” by Treasure et al., which discusses the high mortality, sociocultural causes, and new therapies (e.g., digital interventions) for eating disorders and points out gaps in early screening, comorbidity management, and intervention in current care systems (1). Regarding author productivity, Le Grange Daniel has the most publications (n=61), and Micali Nadia is the most influential author (centrality = 0.28). They are key figures in this field, and future research can build on their work for deeper exploration.

### 4.2 Analysis of research hotspots and trends in adolescent eating disorders

Based on keyword co-occurrence, clustering, and burst analysis, this study highlights the complex interplay between etiology, risk factors, clinical evaluation, management, and comorbidities in adolescent eating disorders. It underscores the need for a multi-faceted, multi-step, and multi-disciplinary approach in their research and management. The analysis shows that comorbidities, etiology, risk factors, assessment, and intervention for adolescent eating disorders are key focuses of academic attention.

#### 4.2.1 Comorbidity

Comorbidity of eating disorders with other mental illnesses is a key focus in adolescent eating disorder research. Eating disorders often coexist with mental illnesses such as mood disorders, anxiety disorders, and personality disorders (38, 39). Adolescents with comorbid conditions tend to exhibit more severe psychiatric symptoms, and the interplay of potential mechanisms underlying these comorbidities may be an important factor leading to poor prognosis. Eating disorders can alter an individual’s cognitive state, causing an excessive preoccupation with eating and thus triggering anxiety. This not only increases the risk of developing psychological problems such as depression and anxiety but also heightens the likelihood of negative behaviors like suicidal ideation (8, 40). A study by Patel et al. (41) found that adolescents with comorbid eating and psychiatric disorders are much more likely to exhibit negative behaviors, especially suicidal ideation, compared to those with only psychiatric disorders. Depression and anxiety, as risk factors for eating disorders, can easily trigger eating symptoms. Adolescents face significant pressures during puberty, including concerns about body image (42), peer acceptance (43), and societal aesthetic standards (44), all of which often exacerbate the severity and persistence of these disorders (45). Moreover, a bidirectional relationship exists between eating disorders and these other conditions, with anxiety and mood disorders potentially preceding the development of eating disorders. For example, one study found that adolescents showing depressive symptoms in the first year of junior high school were at higher risk of developing eating disorder symptoms within the next 12 months (46). This bidirectional relationship complicates diagnosis and treatment because multiple underlying psychological issues need to be considered simultaneously during the therapeutic process. The diverse clinical manifestations of adolescent eating disorders, compounded by comorbid psychological problems such as depression and anxiety, further increase the complexity of diagnosis and treatment. Focusing solely on the eating disorder while neglecting underlying psychological issues often fails to achieve satisfactory therapeutic outcomes and may even lead to relapse. Therefore, future research and practice should place greater emphasis on early identification and intervention, which is of great significance for mitigating the long-term negative impact of eating disorders on adolescents’ physical, psychological, and social development.

#### 4.2.2 Etiology and risk factors

Etiology and risk factors are key research focuses in the field of adolescent eating disorders. Researchers have long explored the causes of eating disorders from a neurophysiological perspective. For instance, one study indicated that eating disorders may be linked to serotonin receptor (5-HT2AR) and brain-derived neurotrophic factor (BDNF) genes (47). Da Luz Neto et al. (48) found an association between eating disorders and hypothalamic-pituitary-adrenal (HPA) axis dysfunction. Notably, a significant relationship exists between eating disorders and diabetes, particularly type 1 diabetes mellitus (T1DM). Adolescents with T1DM, due to the strict dietary control, weight fluctuations from insulin use, and the stress of constant blood glucose monitoring, are highly susceptible to body image dissatisfaction, excessive weight anxiety, and loss-of-control eating behaviors, which considerably increases their risk of developing eating disorders (49). In recent years, researchers have identified relevant factors from multiple perspectives, including gender, BMI, family, personality traits, psychological aspects, and environmental factors (50–52). Body image dissatisfaction (53), low self-esteem (54), emotional dysregulation, and impulsivity (55) are considered major risk factors for eating disorders. Moreover, environmental and sociocultural factors hold significant weight in eating disorder research. Factors such as the impact of sexual and gender minorities (56), social media use (57), and cultural beauty standards can influence adolescents’ mental health and behaviors in various ways. Therefore, a comprehensive understanding of the complex interplay between biological, psychological, and social factors is fundamental to developing effective preventive and personalized treatment strategies. Future research urgently needs to integrate multidimensional data to construct precise risk prediction models and to develop targeted intervention programs based on the individual needs of adolescents to enhance therapeutic efficacy.

#### 4.2.3 Assessment

As a psychiatric disorder associated with high mortality, adolescent eating disorders are significantly linked to severe psychological distress, impaired social functioning, and multiple comorbidities. Despite their prominent clinical impact, global health systems generally struggle with low identification rates and substantial treatment gaps (58, 59). Multiple studies have emphasized the importance of integrating psychological, physiological, and family assessments during the diagnosis of eating disorders. Psychological assessment is a key component in the evaluation of adolescent eating disorders. Commonly used psychological assessment tools include the Eating Disorder Examination Questionnaire (EDE-Q) (60), the SCOFF questionnaire, and the Eating Attitudes Test (EAT-26). Among these, the EDE-Q and the SCOFF questionnaire have been culturally adapted and standardized for screening purposes in many countries (61–63). The Eating Disorder Examination (EDE) is a brief self-report tool that is crucial in identifying abnormal eating behaviors, body image disturbances, and cognitive distortions associated with adolescent eating disorders. It can be used in schools or for large-scale screening. Studies have shown that the EDE performs well in detecting cases and ruling out non-cases of more common eating disorders in primary care settings (64). In addition, medical assessment plays a vital role in adolescent eating disorders. Given that adolescents are at a critical stage of physical development, eating disorders can have a significant impact on their physical health, including malnutrition, electrolyte imbalances, delayed growth, and delayed puberty (65). Given these risks, comprehensive and ongoing medical assessment is crucial for addressing the severe physical consequences of eating disorders. Family dynamics also play a key role in the development, maintenance, and recovery from eating disorders, underscoring the importance of family assessment. Research has confirmed that family functioning is closely linked to the occurrence of eating disorders. Factors such as excessive emotional enmeshment, lack of boundaries, overprotection, or control within the family can influence individual mental health and behavioral patterns (66). Conducting family assessments and identifying potential family dysfunction are essential for developing effective interventions and promoting patient recovery. Family-based assessment can improve treatment outcomes, particularly in adolescents with anorexia nervosa (67). Therefore, the assessment process must be comprehensive and personalized, integrating psychological, medical, and family assessments to fully understand the severity of the disorder and its impact on adolescent health.

#### 4.2.4 Intervention

Visual analyses show that interventions for adolescent eating disorders will be a key future research focus. Current psychotherapies include family-based treatment (FBT) (68), interpersonal psychotherapy (69), cognitive-behavioral therapy (CBT) (70), and dialectical behavior therapy (DBT) (71), all of which are evidence-based and effective for treating adolescent eating disorders. However, research is ongoing to explore innovative strategies to improve treatment outcomes further. Early interventions, especially those combining psychotherapy and nutritional rehabilitation, have proven effective in preventing the progression of eating-disordered behaviors. They can significantly reduce disease severity, slow chronicity, and improve long-term prognosis (72). Meanwhile, the emergence of pharmacological treatments and digital health tools has greatly enriched care options for adolescents with eating disorders, enhancing treatment accessibility and availability (73). While pharmacological intervention isn’t the first-line treatment for eating disorders, it’s indispensable for severe or chronic cases in adolescents, especially those with comorbid depression, anxiety, or OCD (74, 75). Research indicates that Selective Serotonin Reuptake Inhibitors (SSRIs) can enhance the efficacy of CBT for adolescents with depressive or anxious symptoms (76). Fluoxetine, for instance, has been proven to reduce binge-eating episodes in adolescents with bulimia nervosa and alleviate associated emotional distress (77). Furthermore, emerging digital interventions complement traditional eating disorder treatments. Digital platforms enable real-time monitoring, personalized feedback, and improved treatment continuity, bridging the gap in eating-disorder care for rural and remote adolescents (73). Integrating telehealth and digital tools with traditional treatments could pave the way for personalized and continuous care models, enhancing recovery prospects (78). Future research is likely to focus on optimizing digital interventions, investigating their long-term effectiveness, and exploring their integration into conventional treatment plans.

In recent years, eating disorder research has expanded beyond psychiatry to incorporate psychology, sociology, and other disciplines, with interdisciplinary studies on the rise. Concepts like “emotion management”, “mental health”, and “social media” have gained attention. Research confirms a strong link between emotion management and eating disorders, suggesting that effective emotion-management strategies could offer additional support for eating-disorder patients (79, 80). Other studies indicate that social-media use may be associated with eating disorders in adolescents, possibly due to prevalent aesthetic standards and information spread on these platforms (81). With advancing science and technology, interdisciplinary exchange and cooperation will likely play an increasingly important role in adolescent eating disorder research, driving more innovative outcomes in the field.

## 5 Limitations

This study has several limitations listed as follows. Firstly, the coverage and update frequency of different databases vary upon the time of publication, even though we selected the WoSCC database as the source of literature, which may fail to include all studies related to adolescent eating disorders. Secondly, English-language literature was involved in the current study, introducing a selective bias in the current literature. Thirdly, even though CiteSpace and VOSviewer are professional bibliometric tools that can assist us in conducting objective analyses, subjectivity is still difficult to avoid during interpretation due to differences in perspectives among researchers. Fourthly, the time scope was limited to January 1, 2011 - December 31, 2024, so articles published after this period are not included. In light of these limitations, future research can utilize diverse data resources, including multiple databases, languages, a longer period, and a variety of data analysis tools, to gain more comprehensive and accurate results.

## 6 Conclusions

Overall, the annual number of publications on adolescent eating disorders is rising year on year, which indicates that the issue is receiving increasing global attention and attracting more researchers to the field. The visualization analysis using CiteSpace and VOSviewer has revealed the close collaborative networks among countries, institutions, and scholars, as well as the citation patterns of relevant journals and documents in this field. By conducting co-occurrence, clustering, and burst analysis of keywords, we have further mapped out the research trajectory, potential trends, and evolution paths in this area. Current research focuses on the links between eating disorders and other mental illnesses in adolescents, the causes and risk factors of such disorders, and their assessment. Future research will probably concentrate on long-term outcomes of interventions, optimizing personalized assessment and treatment, and applying integrated interdisciplinary treatment frameworks.

## Data Availability

The original contributions presented in the study are included in the article/supplementary material, further inquiries can be directed to the corresponding author/s.
